# Plant Growth Promoting Rhizobacteria, Arbuscular Mycorrhizal Fungi and Their Synergistic Interactions to Counteract the Negative Effects of Saline Soil on Agriculture: Key Macromolecules and Mechanisms

**DOI:** 10.3390/microorganisms9071491

**Published:** 2021-07-13

**Authors:** Alka Sagar, Parikshita Rathore, Pramod W. Ramteke, Wusirika Ramakrishna, Munagala S. Reddy, Lorenzo Pecoraro

**Affiliations:** 1 Department of Biotechnology, Meerut Institute of Engineering and Technology, Meerut 250005, India; alka2011sagar@gmail.com or; 2 Department of Biochemistry, Central University of Punjab, Bathinda 151401, India; pari.rathore1@gmail.com; 3 Faculty of Life Sciences, Mandsaur University, Mandsaur 458001, India; 4 Department of Entomology & Plant Pathology, Auburn University, Auburn, AL 36849, USA; prof.m.s.reddy@gmail.com; 5 School of Pharmaceutical Science and Technology, Tianjin University, Tianjin 300072, China

**Keywords:** salinity, bacteria, fungi, ACC deaminase, green agriculture

## Abstract

Soil saltiness is a noteworthy issue as it results in loss of profitability and development of agrarian harvests and decline in soil health. Microorganisms associated with plants contribute to their growth promotion and salinity tolerance by employing a multitude of macromolecules and pathways. Plant growth promoting rhizobacteria (PGPR) have an immediate impact on improving profitability based on higher crop yield. Some PGPR produce 1-aminocyclopropane-1-carboxylic (ACC) deaminase (EC 4.1.99.4), which controls ethylene production by diverting ACC into α-ketobutyrate and ammonia. ACC deaminase enhances germination rate and growth parameters of root and shoot in different harvests with and without salt stress. Arbuscular mycorrhizal fungi (AMF) show a symbiotic relationship with plants, which helps in efficient uptake of mineral nutrients and water by the plants and also provide protection to the plants against pathogens and various abiotic stresses. The dual inoculation of PGPR and AMF enhances nutrient uptake and productivity of several crops compared to a single inoculation in both normal and stressed environments. Positively interacting PGPR + AMF combination is an efficient and cost-effective recipe for improving plant tolerance against salinity stress, which can be an extremely useful approach for sustainable agriculture.

## 1. Introduction

Plant growth and yield are decreased by soil saltiness, which stands out amongst the basic natural factors [1]. The unbalanced utilization of manures, the use of saline water in the water system, and desertification increase the saltiness of cultivable soils [2]. The saltiness of arable terrains is a noteworthy issue in agribusiness. It causes a critical loss of yield profitability every year [3,4]. According to the FAO, 20% of the world’s irrigated and 2% of dry lands have been affected by salinity [5]. Around 0.3–1.5 million ha of farmland have turned into wasteland due to salinity. Saline soil has electrical conductivity (EC) of 4 dS m^−1^ (corresponding to 40 mM NaCl), resulting in an osmotic pressure of 0.2 MPa, thereby decreasing the yield [4]. Sodium aggregation prompts chlorosis and rot, and changes different physiological processes that bring about diminished yield due to ion toxicity, nodulation, and reduced nitrogen content in plants [6]. The saltiness obstructs root development, resulting in reduced weight of plant parts [7,8].

Soil salinity leads to reprogramming of soil microbial community structure. The beneficial microbiota, plant growth promoting rhizobacteria (PGPR), and arbuscular mycorrhizal fungi (AMF), which occur naturally in the soil and those introduced to combat salinity stress play a key role in the survival of plants [9,10]. This review focuses on the role and mechanisms employed by PGPR, AMF, and their synergistic combination to ameliorate salinity stress.

## 2. Effect of Soil Saltiness on Plant Development

Excessive salt concentrations in the soil affect plant survival by upsetting cell homeostasis and uncoupling major biochemical and physiological processes [1]. The two ions Na^+^ and Cl^−^ in excess harm plant cells through oxidative pressure and osmotic pressure [11]. A typical plant reaction to salt pressure is frequently identified by a low K^+^/Na^+^ proportion in the plant [12]. Plants adapted for growth under saline conditions can abridge sodium harmfulness by restricting Na^+^ uptake, reguiding Na^+^ from shoots to roots, and expelling Na^+^ loadings from root cells [13,14,15]. Further, the hydraulic conductivity and sequestration of toxic Na^+^ particles, amassing of osmolytes, holding higher stomatal conductance, and photosynthetic exercises in plants are expanded in the presence of salt pressure [5,12]. An antagonistic impact of saltiness on complex associations among morphological, physiological, and biochemical procedures include seed germination, plant development, and water and supplement uptake [16,17]. Saltiness additionally influences the developmental proteins, lipid digestion, and photosynthesis [18]. Overall, nutrient deficiency, decrease in osmotic pressure, and reduced water uptake from the soil are the main consequences of soil saltiness.

## 3. Plant Growth Promoting Bacteria

The tight zone of soil, encompassing the root framework, is known as the rhizosphere [19,20]. The term ‘rhizobacteria’ represents bacteria in the rhizosphere, which colonize the root surroundings [19]. Rhizobacteria are important for maintaining the richness of soil as they are fundamental specialists in reusing soil supplements [21]. The plants inoculated with 2–5% of rhizobacteria recorded improved growth, hence the name, plant growth promoting rhizobacteria, PGPR [22]. They include evolutionarily diverse microbes that have the exceptional ability to enhance growth and yield of numerous crops and wild plants [23]. These helpful microorganisms colonize the rhizosphere of plants and increase plant growth and development through different mechanisms [2,24,25].

One potential approach to diminish negative ecological effects that occurred because of the utilization of concoction of manures, herbicides, and pesticides is to use PGPR. PGPR promote the development of plants, sequestration of substantial metals, and counteract the negative effects of pesticides, thereby helping in bioremediation of polluted soils [26,27]. The utilization of PGPR in agribusiness began in the 1950s, and their formulations are available commercially as biofertilizers and biopesticides [28]. PGPR have provided better financial returns because of their capacity to improve seed germination rate and increase crop development and yield of crops [27,29].

### 3.1. Classification and Mode of Action of PGPR

PGPR are classified into extracellular (ePGPR), i.e., existing in the rhizospheric soil or in the intercellular space among root cortex cells, and intracellular (iPGPR), existing inside root cells, mostly in nodular structures. The extracellular PGPR include *Agrobacterium*, *Arthrobacter*, *Azotobacter*, *Azospirillum*, *Bacillus*, *Burkholderia*, *Caulobacter*, *Chromobacterium*, *Erwinia*, *Flavobacterium*, *Micrococcus*, *Pseudomonas,* and *Serratia* [30]. The intracellular PGPR include *Allorhizobium*, *Azorhizobium*, *Bradyrhizobium*, *Mesorhizobium,* and *Rhizobium* of the family Rhizobiaceae [31,32].

**Figure 1:**
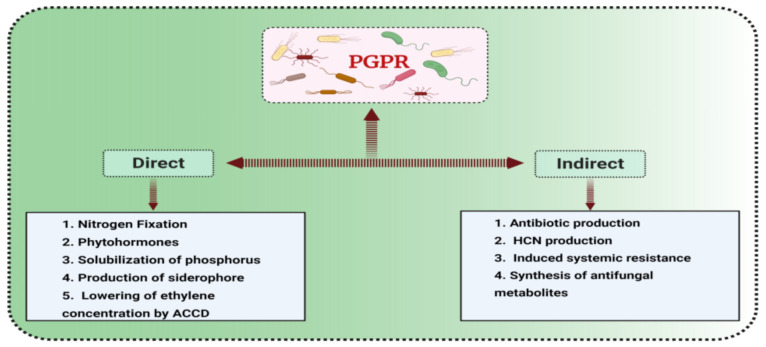
PGP traits of bacteria. Traits that have direct effects and those that have indirect effects (suppression of diseases) on plant growth are shown in the figure.

Numerous studies have shown the different mechanisms of action of PGPR and their applications in agriculture [33]. The generation of phytohormones by PGPR enhances plant growth [34]. PGPR also enhance plant growth through the production of siderophores [35], phosphorus solubilization [36], nitrogen-fixing [37], and lowering ethylene levels in plants through 1-amino-cyclopropane-1-carboxylate (ACC) deaminase, which hydrolyzes ethylene (Figure 1).

Plant diseases reduce plant growth and development under both normal conditions and abiotic stress. ACC produced by PGPR [38,39] diminishes disease by enhancing the production of molecules involved in biocontrol [33]. For example, hydrogen cyanide (HCN); 2,4-diacetylphloroglucinol (DAPG) [40]; and anti-toxins, e.g., phenazine [41] indirectly promote plant survival and fitness under normal conditions and salinity stress.

### 3.2. PGPR as a Major Player in Crop Production Enhancement under Salinity Stress

**Figure 2:**
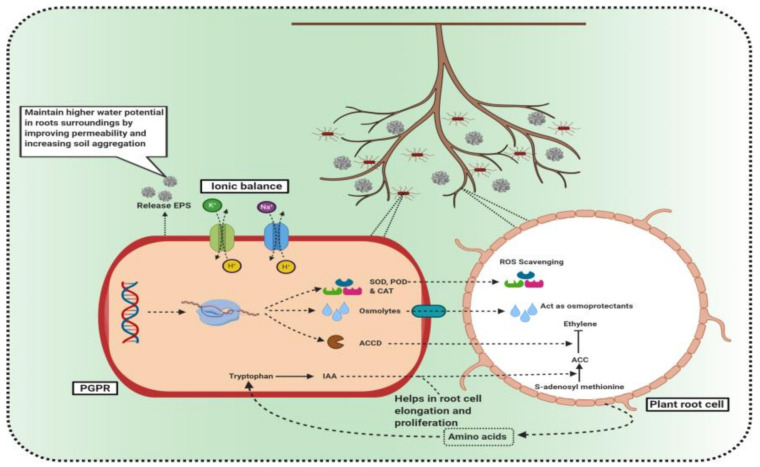
PGPR-mediated salt tolerance by multiple rhizospheric interactions in soil. (1) Release of plant growth regulators to improve nutrition uptake. (2) Production of antioxidant enzymes. (3) Maintenance of ionic homeostasis via transporters. (4) Increased water uptake by improving permeability and soil aggregation through EPS production. (5) Production of osmolytes such as proline and glycine that act as osmoprotectants. (6) Inhibition of ethylene production to reduce stress levels in the plant.

The positive impact of PGPR on harvest yield under biotic and abiotic stresses has prompted the overall utilization of PGPR as biofertilizers for numerous years [28,42,43]. Plant adjustment to saline stress is accomplished by the nearness of the assorted gathering of root-associated microorganisms, which are part of biofertilizers and/or present naturally in the soil. PGPR modify the endogenous hormonal status of the plant, thereby improving the salt resilience of plants [44,45,46]. PGPR, which can live under salinity stress, synthesize and release different plant growth hormones and regulators that significantly promote plant growth, including indole acetic acid (IAA], cytokinin, abscisic acid (ABA), ACC deaminase, trehalose, volatile organic compounds (VOCs), and exopolysaccharides (EPS) [47,48] (Figure 2). Several examples where PGPR enhanced plant growth and yield-related parameters and biofortification under salt stress are shown in Table 1.

PGPR modulate plant gene expression providing better tolerance by improving a plant’s ability to respond to salt stress. This is accomplished by increasing the production of plant metabolites such as betaine, proline, and trehalose, and antioxidant enzymes such as SOD and CAT that scavenge reactive oxygen species [69]. Other beneficial PGP traits such as phosphate solubilization activity and synthesis of siderophores not only confer stress tolerance to plants but contribute to a plant’s overall health by enhancing nutrient uptake [70]. Although PGPR are used as inoculants for biostimulation, biocontrol, and biofertilization [71,72] to facilitate plant growth of many cereals and other important agricultural crops, they can also improve the growth and yield under saline conditions [73,74,75,76].

### 3.3. ACC Deaminase Production by PGPR as a Weapon to Fight Salt Stress

PGPR harboring *acdS* gene encoding ACCD enhance plant growth and development by diminishing plant ethylene synthesized due to salinity stress [77]. ACCD hydrolyzes ACC (precursor of ethylene biosynthesis in higher plants) into alkali and α-ketobutyrate for use as a nitrogen source [78] and enhances plant growth under saline conditions [79,80]. Likewise, ACCD can protect plants from pathogenic microorganisms and drought stress.

ACCD is a multimeric enzyme with a monomeric subunit atomic mass of roughly 35–42 kDa. ACCD uses pyridoxal 5-phosphate as a cofactor [81]. Pyridoxal phosphate is firmly bound to the protein with roughly one particle for every subunit resulting in pyridoxaldimine with absorbance at 418 nm. While a few D-amino acids, D-serine, and D-cysteine can act as substrates for ACC deaminase (less proficiently than ACC), L-serine and L-alanine are aggressive inhibitors of the enzyme [82]. Their substrate ACC is plant-produced but the enzyme is located in the cytoplasm of the microorganism that produces it.

**Figure 3:**
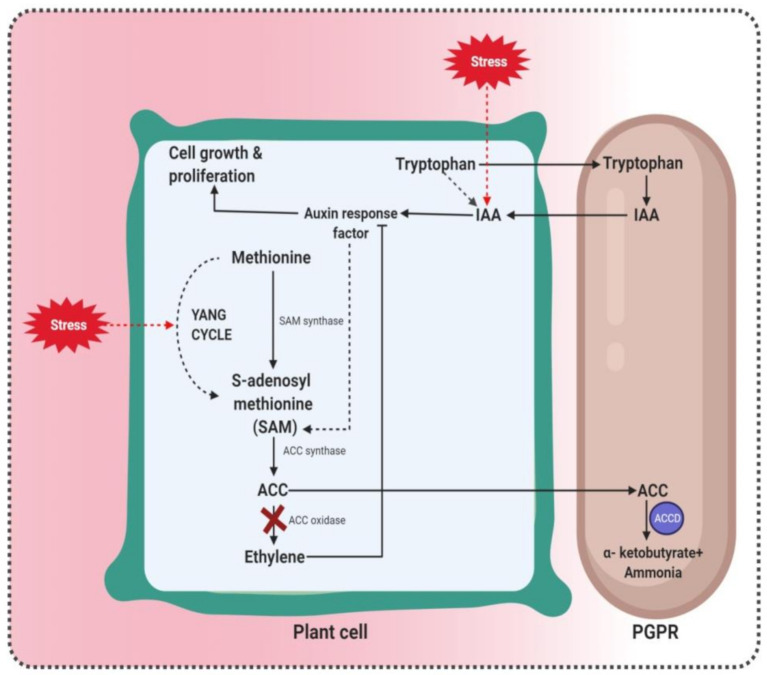
Salt stress increases ethylene production, thereby reducing plant growth. ACC deaminase of PGPR inhibits ethylene biosynthesis under salt stress. PGPR harboring ACC deaminase reduce ethylene production by converting ACC into α-ketobutyrate and ammonia. Adapted from del Carmen Orozco–Mosqueda et al. (2020).

The microbes reduce plant ethylene levels, thereby enhancing plant growth and development, particularly under stressful conditions. This leads to an increase in the root surface area for efficient interaction with soil microscopic organisms and the release of exudates. The established PGPR in association emit IAA, which is taken up by the plant. IAA promotes plant cell expansion and lengthening, and incites ACC synthase to deliver ACC [83] A portion of the plant’s ACC is excreted alongside other macromolecules, for example, sugars, natural acids, and amino acids. The exudates might be used by the rhizospheric microscopic organisms as a nutrient source. ACC is released along with other root exudates. The action of ACCD generates ammonia and α-ketobutyrate, mixes that are additionally processed by the microorganisms (Figure 3).

The plants produce more ACC than needed and furthermore, invigorate the exudation of ACC from the plant, some of which may happen as an outcome of enhanced plant cell division brought about by bacterial IAA [38]. Accordingly, plant growth promoting microbes are provided with a one-of-a-kind wellspring of nitrogen due to ACC that empowers them to multiply under conditions in which other soil microscopic organisms may not promptly thrive. As ACC deaminase acts as a sink for ACC and brings down ACC levels inside the plant, the inhibition of plant growth and development by ethylene (particularly amid times of stress including salinity stress) is diminished, and these plants, for the most part, have longer roots and shoots and greater biomass. Some examples of PGPR with 1-aminocyclopropane-1-carboxylic deaminase (ACCD) activity that survive under salinity stress are given in Table 2.

## 4. Arbuscular Mycorrhizal Fungi (AMF) as Complementary Microorganisms to PGPR to Overcome Salinity Stress

Mycorrhiza is known to be a symbiotic association between fungi and vascular plants, at root level. Arbuscular mycorrhizal fungi (AMF) are obligate mycorrhizal partners that form a beneficial symbiotic association with the roots of over 80% terrestrial plant species, including halophytes, hydrophytes, and xerophytes. AMF are endomycorrhizal fungi (the hyphae of fungi penetrate the cell wall and invaginate the cell membrane) that belong to the phylum Glomeromycota [111]. AMF form vesicles, arbuscules, and hyphae in the associated roots, and produce spores and hyphae in the rhizosphere. The development of a hyphal network by the AMF, which is connected with plant roots, provides plants greater access to soil surface area, resulting in improved growth [112,113]. AMF boost plant nutrition by increasing the availability and translocation of various nutrients. They secrete a proteinaceous compound, glomalin, which helps soil aggregation and stimulates nutrient cycling. AMF play a vital role in improving soil quality and, ultimately, plant health [114].

A number of research studies have reported the ability of AMF to improve plant growth and yield under salinity stress (Table 3). They are known to promote salinity tolerance by employing several mechanisms, such as enhancing water use efficiency and nutrient acquisition by producing plant growth hormones and regulators, improving photosynthetic rate, balancing ionic equilibrium, and producing antioxidants [16,115,116,117,118].

### 4.1. Mechanisms Employed by AMF for Salt Stress Amelioration

#### 4.1.1. Increased Mineral Nutrition

A high concentration of Na^+^ and Cl^−^ in the soil solution competes with the uptake of vital ions such as Ca^2+^, P, K^+^, Mg^2+^, and N, and alters the ideal salt ratios in the soil solution, thereby affecting plant nutrient acquisition and restricting plant growth and biomass. Increased absorption of P via the mycorrhizal fungi contributes most to improve plant growth under salt stress [141]. However, other metabolic processes such as enhanced N assimilation and absorption of other nutrients such as N, K, and Mg seem to be involved in alleviating the deleterious effects of salinity [114]. AMF-plant symbiosis has been demonstrated to increase salinity tolerance in various host plants such as wheat, alfalfa, maize, and tomato (Table 3).

#### 4.1.2. Enhanced Water Uptake

AMF are known to improve the water absorption capacity of plants, due to the network expansion of extraradical hyphae in the soil that pulls more water, making it available to the plant. In addition, AMF induce major changes in the relative abundance of organic solutes by modifying the composition of carbohydrates and inducing accumulation of specific osmolytes such as proline, glycine, and betaine, thus facilitating osmotic adjustment [142]. Furthermore, AMF are able to enhance the functioning of water channel proteins, aquaporins, by modulating their expression, thereby helping in the transport of water inside the cells and maintaining the cellular osmoregulation [143,144]. GintAQPF1 and GintAQPF2, the two aquaporin genes present in the AM fungus *Glomus intraradices*, were found to be overexpressed under osmotic stress conditions, making the fungus tolerant to stress and increasing water supply to the host plant [145].

#### 4.1.3. Ionic Homeostasis

Under saline conditions, the high Na concentration negatively interferes with transporters located in the root plasma membrane, such as K^+^ selective ion channels. As a result, the uptake of mineral nutrients (N, P, K, Fe, Cu, and Zn) is reduced. The high Na^+^/K^+^ ratio interrupts various enzymatic processes and protein synthesis. AMF have been shown to improve the absorption of K^+^, which helps the plants to maintain a lower Na^+^/K^+^ ratio and ionic equilibrium and improve N, P, K, Cu, Fe, and Zn content [146], thereby preventing damage to normal cellular enzymatic processes.

AMF can regulate the movement of excess Na^+^ ions from cells through Na^+^/H^+^ plasma membrane antiporter via modulation of SOS (salt overly sensitive) genes, thus maintaining ion homeostasis. For instance, the AMF associated with *Oryza sativa* have been shown to regulate the expression of genes encoding transporters, i.e., *OsSOS1*, *OsNHX3*, *OsHKT2;1*, and *OsHKT1*, which are involved in maintaining ion homeostasis, thereby improving plant tolerance to salinity [147].

#### 4.1.4. Phytohormone Synthesis

The AMF produce auxins and cytokinins (CKs) that help in the growth and development of the plant and also stimulate the synthesis of these hormones in plants under stress [148]. Plants associated with AMF show enhanced synthesis of abscisic acid (ABA), jasmonic acid (JA), and salicylic acid (SA) that act as signal molecules during the process of AMF symbiosis [113,116,149]. Modulation of phytohormone synthesis by AMF confers drought and salt tolerance in plants [150].

#### 4.1.5. Improved Photosynthesis

Salinity stress decreases photosynthesis by reducing chlorophyll content and photosynthetic enzymes activity. This is due to the reduction in the uptake of Mg^+^ that is needed for chlorophyll biosynthesis. Increased absorption of Mg^+2^/Na^+^ via AMF contributes to the regulation of plant photosynthesis under salinity stress [151]. The symbiotic association of plants and AMF upregulate the expression of chloroplast genes *RppsbA* and *RppsbD* during salt stress [152]. This results in higher PSII efficiency and enhanced photosynthetic capacity. *Glomus mosseae* inoculation significantly increased leaf chlorophyll content in peanut plants under salinity stress [153]. Similarly, tomato plants treated with salt exhibited a higher amount of chlorophyll a and b, total chlorophyll content, and carotenoid content after inoculation with AMF [154].

#### 4.1.6. Antioxidant Production

AMF facilitate plants to modulate salinity stress by increasing the activities of antioxidant enzymes such as catalase (CAT), superoxide dismutase (SOD), peroxidase (POD), ascorbate peroxidase (APX), glutathione reductase (GR), monodehydroascorbate reductase (MDHAR), and dehydroascorbate reductase (DHAR), and glutathione-S-transferase that protect plants from oxidative damage [112,146,149,155]. These enzymes help to alleviate the excess ROS and maintain the equilibrium of the formation and removal of ROS, providing the host plant better tolerance against oxidative stress.

## 5. Co-Inoculation of AMF and PGPR Can Mitigate the Effects of Salinity in Plants

**Figure 4:**
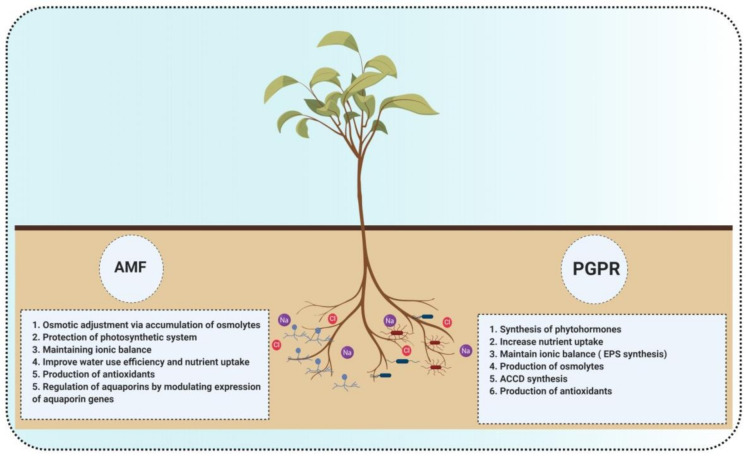
PGPR and AMF mechanisms for salt stress amelioration in plants: common and different mechanisms used by PGPR and AMF to combat salt stress.

The coexistence of PGPR and AMF in the rhizosphere is very beneficial for the growth and development of most plants. This synergistic effect is a result of positive interactions between PGPR and mycorrhizal fungi that help promote the growth of each other, which ultimately benefits the plant [156]. For example, PGPR enhanced AMF growth and survival by affecting root colonization and nutrient uptake [157]. The synergistic interactions between PGPR and AMF were also observed in plants exposed to the saline environment [158]. Combined inoculation of AMF with other PGPR exerted positive effects on the growth of several crop plants. These include enhanced production of soluble sugars, organic acids, antioxidant enzymes, and compounds for ROS scavenging, and reducing Na^+^ levels in plants subjected to salt stress. In addition, upregulation of sodium ion channels, ABA-signaling, and salt overly sensitive (SOS) pathway mediate superior plant performance under a saline environment [159]. The initial plant response to salinity in the presence of PGPR and AMF is characterized by enhanced phytohormone synthesis and accumulation of osmoprotectants followed by Na^+^ export outside the cell via HKT transporter. The synergistic interaction of AMF and PGPR may upregulate the expression of HKT and Na^+^/H^+^ antiporter genes. Thus, the dual inoculation of PGPR and AMF could be an effective tool for alleviating salt stress in crops (Figure 4).

Table 4 shows examples of the beneficial interaction between PGPR and AMF to boost plant growth. The efficacy of co-inoculation of AMF and PGPR have been shown in sorghum [160], wheat [161,162], swamp oak [163], bean [164], and watermelon [146], and several other plants to promote growth and/or improve stress tolerance. Although an increase in plant growth and grain yield was observed when PGPR and AMF are used in combination, several factors such as environmental conditions, soil quality, and the microbial strains used, contribute to variable results. For example, a 128% increase was observed in combined grain yields of finger millet and pigeon pea in intercropping conducted at the Kolli Hills site but not the Bangalore site [165]. Generally, an increase in crop yield of approximately 30–40% was observed in combined PGPR and AMF inoculation in field studies. Co-inoculation of *Rhizobium* with AMF resulted in significant enhancement of yield, nodulation, leghemoglobin, nitrogenase activity, IAA synthesis, and nutrient uptake of alfalfa subjected to salinity stress [166]. Inoculation of soybean with AMF improved various attributes as observed in alfalfa, but also conferred protection against membrane damage by reducing hydrogen peroxide and lipid peroxidation [167]. Morphological and genetic level approaches to study genes associated with metabolism, nitrogen fixation, and cell colonization events revealed the occurrence of nutritional exchanges between endobacteria, fungi, and plants. Some AMF species produce metabolites such as organic acids, volatile compounds (ethylene), and nonvolatile compounds that attract specific bacteria [160]. Similarly, some of the bacteria known to enhance colonization of AMF are referred as mycorrhiza-helper bacteria (MHB). PGPR solubilize phosphates in soil whose absorption is enhanced by effective colonization of AMF [168]. ACC deaminase production by PGPR enhances their symbiotic interaction with AMF due to reduced ethylene levels [169]. Plant roots associated with AMF showed lower ethylene and higher JA levels [170]. PGPR and AMF enhance ABA, which regulates stomatal closure and plant growth through the ABA-signaling pathway during salinity and drought stress [171,172]. The expression of phosphate transporter genes was also upregulated. Cytokinin, isopentenyl adenosine, auxin, IAA, gibberellin A4, and ethylene were observed in the spores of AMF [173]. Wheat root exudates harbor benzoxazinoid metabolites whose production is enhanced by AMF, thereby inducing chemotaxis in PGPR [174]. PGPR and AMF together strengthen host immune response to confer resistance to biotic and abiotic stresses [175]. Callose (β-glucan polysaccharide) is deposited on the cell wall when plants are co-inoculated with PGPR and AMF. Callose deposition under salt stress is mediated by Cys-rich receptor-like kinase 2 [176]. A higher production of malondialdehyde (MDA) under salt stress indicates membrane lipid peroxidation, which is neutralized by PGPR and AMF through scavenging of free radicals [170].

## 6. Conclusions

Salinity stress is a major deterrent to agricultural production. It has devastating effects on plant growth and reproduction, resulting in reduced yield. Plants have an inherent ability to respond to specific types of stress. PGPR play key roles in salt stress tolerance and plant growth promotion, with direct and indirect mechanisms. Plants inoculated with ACC deaminase producing PGPR become tolerant to salt stress. ACC metabolizing bacterial strains promote plant growth, increase root/shoot length, and improve plant biomass under salinity stress by lowering ethylene accumulation. The increase in N content in the rhizosphere of legumes considerably accounts for improvement in nodulation and N-fixing capacity, resulting from cooperative interaction of *Rhizobium* and AMF. PGPR and AMF can colonize the root–soil environment to enhance plant growth, yield, nutrient content, and soil health due to synergistic interactions. This is achieved through the production of phytohormones and antioxidants, ionic homeostasis, and improved photosynthesis under salinity stress. The exploitation of these microbial populations needs a systematic strategy to optimize their potential in enhancing plant tolerance to salt stress. The employment of PGPR and AMF in field conditions has certain limitations such as short shelf life, variability in performance, and effect on the diversity and abundance of soil microbiota based on short term studies. In many instances, the interactions of PGPR and AMF with native soil microbes are not known. Some signaling pathways are common to biotic and abiotic (salinity stress) stress. PGPR evade plant defense systems. These mechanisms, if transmitted to pathogens, can have deleterious effects on plants. A comprehensive understanding of plant–PGPR–AMF–soil interactions would pave the way for efficient utilization of PGPR and AMF to counter salinity stress and foster the next green revolution.

## Figures and Tables

**Table 1:** Role of PGPR in crop/plant improvement under salinity stress.

PGPR	Crop/Plant	Response	Reference
*Bacillus cereus*, *Pseudomonas* species	Rice	Increased N (26%), P (16%), K (31%)	[49]
*Bacillus amyloliquefaciens*	Rice	Increased plant growth	[50]
*Thalassobacillus denorans*, *Oceanobacillus kapialis*	Rice	Increased germination percentage and rate	[51]
*Bacillus subtilis*, *Arthrobacter* sp.	Wheat	Increased dry biomass, total soluble sugars, and proline content	[52]
*Planococcus rifietoensis*	Wheat	Enhanced growth and yield	[53]
*Thalassobacillus*, *Bacillus*, *Halomonas*, *Oceanobacillus*, *Zhihengliuella* sp.	Wheat	Increased the root and shoot length, and plant fresh weight	[54]
*Enterobacter cloacae*	Wheat	Improved growth parameters, biomass, and chlorophyll content	[55]
*Klebsiella* sp.	Wheat	Increased proline, total soluble sugar, and total protein content of treated plants	[56]
*Enterobacter cloacae*	Maize	Increased root and shoot growth	[57]
*Staphylococcus sciuri*	Maize	Enhanced nutrient, chlorophyll, and protein content	[58]
Phosphate solubilizing bacteria	Maize and Peanut	Increased seed germination, plant growth, and P content	[59]
*Curtobacterium flaccumfaciens*	Barley	Increased plant growth	[60]
*P. aeruginosa*, *P. stutzeri*	Tomato	Enhanced root and shoot length	[61]
*Bacillus aryabhattai* H19-1, *B. mesonae* H20-5	Tomato	Significantly higher levels of proline, abscisic acid (ABA), and antioxidant enzyme activities were observed	[62]
*B. arryabhattai* H19-1, *B. mesonae* H20-5	Soybean	Enhanced root and shoot length and dry biomass	[63]
*Sinorhizobium meliloti*, *Paenibacillus yonginensis*	Lucerne and Ginseng	Increased chlorophyll and carotenoid	[64,65]
*A. chroococcum*, *Lactobacillus* sp.	Lettuce	Increased root length at 50 and 100 mM NaCl	[66]
*Enterobacter cloacae*	Canola	Increased proline levels	[67]
*Bacillus*, *Pseudomonas*, *Enterobacter*, *Azotobacter*, *Rhizobium*	Strawberry	Increased plant height	[68]

**Table 2:** Alleviation of the impact of salinity stress by PGPR with ACC-deaminase activity.

PGPR	Crop	Response	Reference
*P. fluorescens*	Rice	Maintained root colonization potential by osmotolerance mechanisms	[84]
*Bacillus*, *Microbacterium*, *Methylophaga*, *Agromyces*, *Paenibacillus*	Rice	Enhanced yield	[85]
*Alcaligenes*, *Bacillus*, *Ochrobactrum*	Rice	Positive impact on germination percentage, shoot and root growth, and chlorophyll content	[86]
*Pseudomonas putida*, *Pseudomonas fluorescens*	Rice	Promoted rice growth by colonizing rice roots	[87]
*Pantoea agglomerans* strain KL	Rice	Increased length, biomass, and photosynthetic pigments	[88]
*Enterobacter cloacae* (KP226569)	Rice	Enhanced seed germination and growth	[89]
*Enterobacter* sp. PR14	Rice and Millets	Enhanced seed germination, root and shoot length	[90]
*P. putida*, *P. aeruginosa*, *S. Proteamaculans*	Wheat	Increased plant height, root length, and grain yield	[91]
*P. putida*, *Enterobacter cloacae*, *Serratia ficaria*, *P. Fluorescens*	Wheat	Improved growth and yield	[92]
*Azospirillum* strains	Wheat	Increased shoot dry weight and grain yield	[93]
*Pseudomonas putida*, *Pseudomonas fluorescens*, *Enterobacter cloacae*, *Serratia ficaria*	Wheat	Enhanced germination rate and improved the nutrient status	[94]
*Bacillus*, *Hallobacillus*	Wheat	Enhanced plant growth	[2]
*Klebsiella* sp.	Wheat	Increased plant biomass and chlorophyll content	[95]
*B. subtilis*	Wheat	Increased growth and yield	[46]
*Bacillus licheniformis*	Wheat	Increased root and shoot length, fresh weight, and dry weight	[96]
*Chryseobacterium gleum* sp. SUK	Wheat	Increased yield	[97]
*Pseudomonas putida* (W2), *P. fluorescens* (W17)	Wheat	Increased growth and yield	[98]
*P. syringae*, *P. bathycetes*, *E. aerogenes*, *F. ferrugineum*, *P. fluorescens*	Maize	Improved growth, yield, and nutrition	[78]
*Pseudomonas syringae*, *Pseudomonas fluorescens*	Maize	Significantly improved yield	[99]
*Enterobacter cloacae*	Maize	Increased seed germination and elongation of root and shoot	[100]
*Enterobacter cloacae* (KP226575)	Millets	Increased seed germination and elongation of root and shoot	[101]
*Pseudomonas syringae*, *Pseudomonas fluorescens*, *Rhizobium phaseoli*	Mung bean	Improved seedling growth and nodulation	[102]
*Rhizobium*, *Pseudomonas*	Mung bean	Improved growth, physiology, and quality of seed	[103]
*Brevibacterium epidermidis*, *Bacillus aryabhattai*	Canola	Increased seed germination	[104]
*Pseudomonas* sp.	Barley and Oats	Enhanced root biomass	[105]
*Aneurinibacillus aneurinilyticus*, *Paenibacillus* sp.	French bean	Enhanced plant growth	[106]
*Paenibacillus mucilaginosus* strain N3	Green gram	Increased overall dry biomass	[107]
*Bacillus megaterium*, *Variovorax paradoxus*	Cucumber	Increased growth	[108]
*Pseudomonas* strain	Groundnut	Increased total yield	[109]
*Leclercia adecarboxylata*	Tomato	Improved plant growth	[110]

**Table 3:** Response of AMF on different plants against salinity stress.

AMF	Crop	Plant Response Under Salt Stress	Reference
*Glomus mosseae*, *G. etunicatum*, *G. intraradices*	Wheat	Significant enhancement of N, K, P, Ca, Mg, Mn, Cu, Fe, Zn uptake	[119]
*Glomus viscosum* H.T. Nicoson strain A6	Alfalfa	Improved K uptake	[120]
*Glomus intraradices*	Carnation	Flower dry weight and the total number of flowers per plant increased; number of buds and flowers increased	[121]
*Glomus intraradices*	Tomato	Na uptake in inoculated plants lower compared to control; AMF plants had greater values for K/Na and Ca/Na in both shoots and roots	[122]
*Glomus mosseae*, *Glomus versiforme*	Orange	Accumulation of ROS and membrane damage reduced; SOD activity was largely induced	[123]
*Glomus mosseae*, *Glomus intraradices*	Olive	AMF colonization was more effective under saline condition; shoot and root dry weight increased; K concentration increased in shoot	[124]
*Glomus intraradices*	Sweet Basil	Reduced Na concentration in plants; treated plants grew faster	[125]
*Glomus clarum*	Pepper	Significantly improved shoot, root dry matter, and fruit yield; improved chlorophyll concentration; proline concentration was lower	[126]
*Glomus mosseae*, *Paraglomus occultum*	Citrus	Leaf number, leaf area, shoot and root dry weights increased; relative water content increased; root concentration of K^+^, Ca^2+^, and Mg^2+^ were higher	[127]
*Glomus etunicatum*, *Glomus intraradices*, *Glomus mosseae*	Cucumber	Increased biomass, photosynthetic pigment synthesis, and antioxidant enzymes	[128]
*Rhizophagus irregularis*	Tomato	Enhanced shoot FW, leaf area, leaf number, root FW, and levels of growth hormones	[129]
*Claroideoglomus etunicatum*	Rice	Improved quantum yield of PSII photochemistry, net photosynthetic rate, and stomatal conductance	[130]
*Claroideoglomus etunicatum*	Indian Walnut	Increased shoot and root dry mass, stomatal conductance, soluble sugars, free α-amino acids, and Na^+^ and K^+^ uptake	[131]
*Glomus intraradices*	Tomato	Improved dry matter, ion uptake, growth parameters, and chlorophyll content	[132]
AMF consortia	Physic nut	AMF lessen the deleterious effect of salt stress (up to 0.5% NaCl) on seedling growth parameters under salt levels	[133]
*Glomus deserticola*	Parwal	AMF improved yield and alleviated deleterious effects of salt	[134]
*Glomus etunicatum*, *G. mosseae*, *G. intraradices*	Wheat	Selection of the right combination of AMF species improved wheat cultivation under salinity stress	[135]
*Glomus mosseae*	Pigeon pea	AMF inoculation increased solute accumulation to maintain osmotic balance and antioxidant enzyme activity under stress	[136]
*Glomus intraradices*	Lettuce	Shoot dry weight and shoot water content increased, and transpiration rate decreased	[137]
*Glomus mosseae*, *Glomus claroideum*, *Glomus intraradices*	Milkvetch	*G. intraradices* performed better than two other fungi in root colonization and enzyme activity; synergistic interaction between fungi under NaCl stress also seen	[138]
*Glomus mosseae*	Maize	AMF symbiosis improved solute accumulation in maize leaves to mitigate the negative impact of soil salinity	[139]
*Glomus fasciculatum*	English beechwood	AMF was very effective in strengthening the tolerance of *Gmelina arborea* grown in arid and semiarid areas	[140]

**Table 4:** Combined effect of PGPR and AMF under salinity stress in different plants.

Plant Species	AMF Partner	PGPR Partner	Application	Ref.
Pigeon pea and finger millet	AMF	*Pseudomonas*	128% yield increase was observed in finger millet and pigeon pea intercropping system at Kolli Hills but not at Bangalore site	[165]
Common bean	*Glomus irradicans*	*Bacillus megaterium*	Enhanced chlorophyll and antioxidant enzymatic activity at all tested salinity levels	[171]
Russian Olive	*Glomus mosseae*	*Bacillus amyloliquefaciens*	Enhanced seedlings growth and improved soil nutrient uptake	[172]
French honeysuckle	*Rhizophagus intraradices*	*Pseudomonas* sp., *Bacillus subtilis*	Soil quality improvement by modulating enzymes involved in the cycling of carbon, nitrogen, and phosphorus	[156]
Talh tree	*Claroideoglomus etunicatum*, *Rhizophagus intraradices*, *Funneliformis mosseae*	*B. subtilis*	Increased plant biomass, nodulation, leghemoglobin, crude protein content, and photosynthetic pigments	[148]
Potato	*Glomus intraradices*, *G. mosseae*	*P. fluorescens* T17-4, *P. fluorescens* VUPf5, *P. fluorescens* F140	Increased fresh and dry weight, other growth factors and chlorophyll	[173]
Maize	*Glomus etunicatum*	*Methylobacterium oryzae* CBMB20	Increased dry biomass, AMF root colonization, and nutrients in plants under salt stress; Na^+^ uptake reduced by 41%	[174]
Potato	*Glomus mosseae*, *G. fasciculatum*	Two strains of *Pseudomonas* (P116 and P173) and *Bacillus* (*Bacillus subtilis* and *B. megaterium*)	Significant effect on chlorophyll index and phosphorus absorption	[175]
Common bean	*Glomus mosseae*	*Pseudomonas florescens*	Increased proline content, CAT, and POX activity	[164]
Cucumber	*Gigaspora rosea* BEG9	*Pseudomonas putida* UW4	Increased leaf area and photosynthetic efficiency	[158]
Lettuce	*Glomus* spp.	*Pseudomonas mendocina*	Enhanced plant biomass	[176]

## Data Availability

Not applicable.
